# The Type B Flagellin of Hypervirulent *Clostridium difficile* Is Modified with Novel Sulfonated Peptidylamido-glycans*

**DOI:** 10.1074/jbc.M116.749481

**Published:** 2016-10-07

**Authors:** Laura Bouché, Maria Panico, Paul Hitchen, Daniel Binet, Federico Sastre, Alexandra Faulds-Pain, Esmeralda Valiente, Evgeny Vinogradov, Annie Aubry, Kelly Fulton, Susan Twine, Susan M. Logan, Brendan W. Wren, Anne Dell, Howard R. Morris

**Affiliations:** From the ‡ Department of Life Sciences, Imperial College London, South Kensington Campus, London SW7 2AZ, United Kingdom,; the ¶ Department of Pathogen Molecular Biology, London School of Hygiene and Tropical Medicine, Keppel Street, London WC1E 7HT, United Kingdom,; the ‖ Vaccine Program, Human Health Therapeutics Portfolio, National Research Council, Ottawa, Ontario K1A 0R6, Canada, and; § BioPharmaSpec, Suite 3.1 Lido Medical Centre, St. Saviours Road, Jersey JE2 7LA, United Kingdom

**Keywords:** bacteria, glycosylation, Gram-positive bacteria, mass spectrometry (MS), nuclear magnetic resonance (NMR), Clostridium difficile, flagellin, modification, sulfonated

## Abstract

Glycosylation of flagellins is a well recognized property of many bacterial species. In this study, we describe the structural characterization of novel flagellar glycans from a number of hypervirulent strains of *C. difficile*. We used mass spectrometry (nano-LC-MS and MS/MS analysis) to identify a number of putative glycopeptides that carried a variety of glycoform substitutions, each of which was linked through an initial *N*-acetylhexosamine residue to Ser or Thr. Detailed analysis of a LLDGSSTEIR glycopeptide released by tryptic digestion, which carried two variant structures, revealed that the glycopeptide contained, in addition to carbohydrate moieties, a novel structural entity. A variety of electrospray-MS strategies using Q-TOF technology were used to define this entity, including positive and negative ion collisionally activated decomposition MS/MS, which produced unique fragmentation patterns, and high resolution accurate mass measurement to allow derivation of atomic compositions, leading to the suggestion of a taurine-containing peptidylamido-glycan structure. Finally, NMR analysis of flagellin glycopeptides provided complementary information. The glycan portion of the modification was assigned as α-Fuc3N-(1→3)-α-Rha-(1→2)-α-Rha3OMe-(1→3)-β-GlcNAc-(1→)Ser, and the novel capping moiety was shown to be comprised of taurine, alanine, and glycine. This is the first report of a novel *O*-linked sulfonated peptidylamido-glycan moiety decorating a flagellin protein.

## Introduction

The intestinal pathogen *Clostridium difficile* is the leading cause of antibiotic-associated diarrhea worldwide. The pathogen colonizes the gastro-intestinal tract when the normal microbiota is disturbed after antibiotic treatment, causing *C. difficile* infection in susceptible patients. In the past decade, *C. difficile* infection mortality has increased dramatically since the emergence of hypervirulent strains, such as the PCR ribotype 027 (RT027)^4^ and RT023 lineages (1, 2).

The reasons for the increase in severity of *C. difficile* infection caused by hypervirulent strains are still not well understood. Molecules implicated in *C. difficile* virulence include the secreted toxins TcdA and TcdB and a variety of cell surface biopolymers (3–6). Among the latter, flagella, which are responsible for the pathogen's motility, are believed to have roles in virulence because disruption of their biosynthesis and expression affects colonization, biofilm formation, and toxin production (7).

The flagellin proteins of *C. difficile* are known to be post-translationally modified with *O*-linked glycans, and there is evidence that glycosylation can affect motility and virulence (8, 9). Flagellin *O*-glycosylation is widespread in Gram-negative bacteria (10) but so far has only been found in three Gram-positive genera, *Clostridium*, *Listeria*, and *Paenibacillus* (11–13). A great deal of diversity exists among flagellin glycans, but there are some common themes. For instance, many Gram-negative flagellins have a single pseudaminic acid or legionaminic acid residue at each of their *O*-glycosylation sites. These sugars can be substituted with a variety of functionalities, such as acyl and acetamido groups, resulting in considerable structural heterogeneity. Interestingly, the first *Clostridium* species to have its flagellin glycosylation characterized, *Clostridium botulinum*, was found to share this type of glycosylation. Thus, its flagellin is substituted with the legionaminic acid derivative 7-acetamido-5-(*N*-methyl-glutam-4-yl)-amino-3,5,7,9-tetradeoxy-d-glycero-α-d-galacto-nonulosonic acid (αLeg5GluNMe7Ac) (12) In contrast, *C. difficile* post-translational modifications appear to be quite different in structural composition from those that have been found in Gram-negative organisms.

The best characterized *C. difficile* flagellin is that from the first strain to have its genome sequenced (14). This PCR ribotype 012 strain (strain 630) was isolated from a Swiss hospital patient in 1982. It is an epidemic, multidrug-resistant strain and predates the emergence of the hypervirulent strains. The 630 flagellin is modified at up to seven sites with the monosaccharide *N*-acetylglucosamine (GlcNAc), which is substituted with a phosphodiester-linked *N*-methyl-l-threonine residue (8, 9). RT027 and RT023 strains, however, appear to lack this unusual amino acid modification. Preliminary data from MS investigations of RT027 flagellins were interpreted as consistent with the presence of HexNAc-linked oligosaccharides up to a pentasaccharide in length. Mass increments in the MS data were attributed to various compositions of deoxyhexose, methylated deoxyhexose, HexNAc, and heptose, but full structures were not defined at that time because of technological limitations (8).

In this study, we have rigorously characterized flagellin glycosylation in several emerging hypervirulent clonal strains, including RT027, RT023, RT106, and RT001. We have discovered several variants of a novel peptidylamido-glycan sulfonate structure decorating hydroxyamino acids (Ser and Thr) in the flagellin (FliC) protein. Here we describe the chemical characterization of this newly discovered structure using Q-TOF technology (15, 16) applied in a variety of structural elucidation strategies (17, 18), including positive and negative ion collisionally activated decomposition (CAD) MS/MS to produce unique and interpretable fragmentation patterns and high resolution accurate mass measurement to allow derivation of detailed atomic compositions. Confirmation and extension of the MS structural interpretations was then made by purifying a number of glycopeptides in sufficient quantities to define each structure by NMR.

## Results

### 

#### 

##### Mass Spectrometric Analysis of Flagellin Glycopeptides

A preliminary structure of RT027 flagellin glycosylation had been studied previously by LC-MS/MS analysis (8), but full structures were not solved due to technical limitations. In the study presented here, following nano-LC-MS and MS/MS analysis, a number of putative glycopeptides derived from RT0207 and other flagellins were identified via a search for oxonium ions (*e.g. m*/*z* 204) in the data sets. This led to the discovery of a number of glycopeptide candidates carrying a variety of glycoform substitutions, including the peptides LLDGSSTEIR, VALVNTSSIMSK, and QMVSSLDVALK. Each of these peptides carried a glycan modification that was linked through an initial HexNAc residue to Ser or Thr, followed by deoxyHex/methyldeoxyHex, methyldeoxyHex/deoxyHex, and methyldeoxyHex/methyldeoxyHex in positions 2/3, respectively. The majority of structural work reported here has concentrated on the LLDGSSTEIR glycopeptides from strain R20291, and interestingly, two variant structures carried on this peptide were present in the LC-MS chromatograms at *m*/*z* 998^2+^ and 991^2+^, where the MS/MS fragmentation pattern clearly showed (in addition to carbohydrate moieties) the presence of a novel structural entity not previously observed in sugar or amino acid chemistry.

**FIGURE 1.**
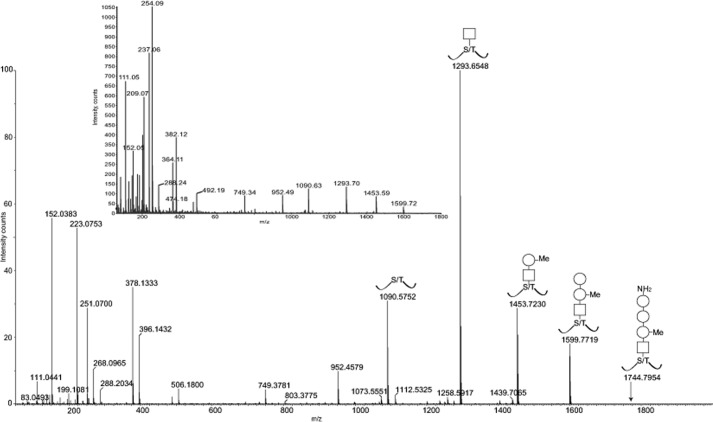
**Positive ion on-line nano-LC MS/MS high resolution CAD mass spectrum of *m*/*z* 998^2+^ (*main spectrum*) with equivalent low resolution MS/MS spectrum of m/z 991^2+^ (*inset*).** Note the low mass signals (below *m*/*z* 400), many of which do not correlate with either known peptide or carbohydrate fragments and which therefore indicated the discovery of novel structural features in these glycopeptides. For full interpretation, see “Results” and Scheme 1.

The CAD MS/MS spectra of *m*/*z* 998^2+^ and 991^2+^ molecules are shown in Fig. 1, with the *main spectrum* showing the high resolution data (to 4 decimal places) for the larger molecule (*m*/*z* 998^2+^), and the *inset* showing the low resolution spectrum of *m*/*z* 991^2+^ for comparison. From these data, it is clear that a HexNAc-methyldeoxyHex-deoxyHex- glycosyl substituent is attached to the peptide backbone (seen at 1090, M + H^+^) via signals at *m*/*z* 1293, 1453, and 1599, respectively (corresponding to glycosidic cleavages), together with a series of y″ ions beginning at *m*/*z* 749 and extending with the same substituents, identically for both precursor ions. A further less intense signal is present at *m*/*z* 1744 in the high resolution data extending the glycosylation sequence by an amino-dideoxyHex unit. The high resolution mass measurement of the 991^2+^ signal compared with the 998^2+^ signal shows that the 14-atomic mass unit mass difference corresponds to a CH_2_ difference between the two structures. The clearly novel aspect of these glycopeptides can be seen in the substantial fragment ions at *m*/*z* 396, 378, 268, 251, 223, and 152, which do not immediately correlate with sugar or amino acid origin. The equivalent signals are present in the 991^2+^ data (*inset spectrum*) 14 Da lower at *m*/*z* 382, 364, 254, 237, 209 except for *m*/*z* 152 (same), showing that the 14-Da mass difference resides between the 152 and 396/382 fragments in the structure. When cross-correlating the observed 396/382 and 1599 signals with the molecular masses observed in the 998^2+^/991^2+^ quasimolecular ions, these fragments are additive to the molecular mass (allowing for hydrogen transfers) and thus represent between them the overall glycopeptide structure, as shown schematically in Fig. 1 for the 998^2+^ variant.

**SCHEME 1.**
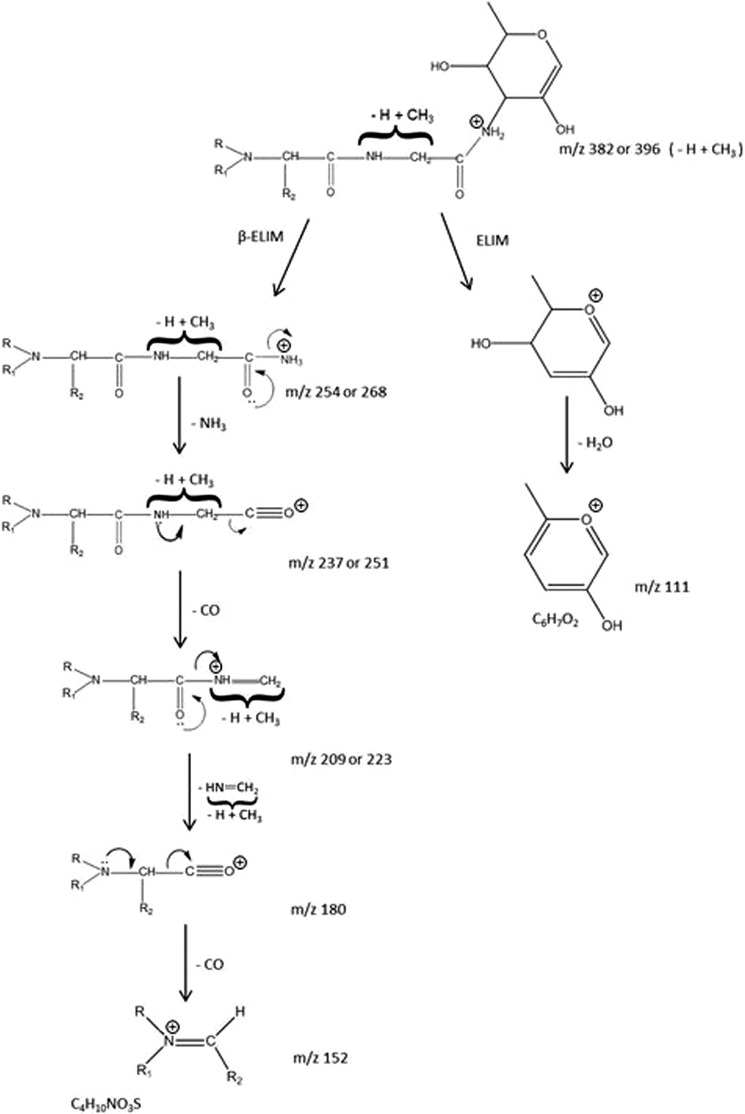
**Summary of the interpretation and mechanistic logic used to assess the mass spectrometric fragmentation data determined in this study (see Fig. 1) showing the probable structural assignments.** Conclusions were aided by the derivation of atomic compositions of key fragments shown in Table 1.

The interpretation of the mechanisms leading to these fragment ions was greatly assisted by the atomic compositions determined from the accurate masses in the high resolution Q-TOF data (see representative data in Table 1) and also by the presence of “counterion” data, as is often observed in doubly charged MS/MS spectra. For example, the aminodideoxyHex residue extension from *m*/*z* 1599 to 1744 must therefore be present in the m/z 396 counterpart, and its partial loss (128 Da) by β-elimination is observed to give *m*/*z* 268 in Fig. 1, whereby the amino function is retained by the 268 ion, which itself then loses first ammonia to *m*/*z* 251 and then carbon monoxide to *m*/*z* 223 from the amide group thus assigned. The charge can of course be retained for a proportion of fragments on the “counterion” instead of *m*/*z* 268, which would be present at *m*/*z* 129 from the elimination mechanism. The significant signal at *m*/*z* 111 (C_6_H_7_O_2_) was assigned to that origin via loss of water to give a highly stable triply conjugated cyclic ion, available without rearrangement by postulating a 3-amino substitution. The next significant ion in the low mass region is seen at *m*/*z* 152, and it was recognized that the observed accurate mass difference of 71.037 Da (223.0753 minus 152.0383), could correspond to either an alanine or its isomer *N*-methyl glycine (atomic composition C_3_H_5_NO, theoretical mass 71.0371). In mechanistic terms, the carbonyl of such a unit could be forming the amide linkage to the amino sugar in *m*/*z* 396, which would predictably fragment to give the 268, 251 (b_2_), and 223 (a_2_) ions and then to give a terminal “b_1_” ion at *m*/*z* 180 (from loss of Ala or *N*(Me)Gly), the signal for which is not observed. However, the *m*/*z* 152 mass is 28 daltons (CO from the atomic composition) below this mass, and it is very common for the a_1_ ion subfragment (an aldimine) to be the more intense ion in peptide fragmentation, particularly if the nitrogen is alkylated by methyl or another grouping giving rise to a tertiary nitrogen. The summary of the interpretation logic used to assign the above MS signals at that stage of the study is shown in Scheme 1.

**TABLE 1:** **Atomic compositions deduced for key signals in the high resolution MS/MS data** This table shows the deduced atomic compositions for the experimental (measured) masses observed for key signals in the high resolution MS/MS data (Fig. 1) together with the theoretical masses of those compositions. Note that certain fragments, such as peptide y″ ions and the peptide quasimolecular ion (M + H^+^) serve as useful internal standards, confirming the mass accuracies across the data set. Key discoveries from these data included the finding of sulfur in the *m*/*z* 152 and higher mass fragments, thus confirming a novel structural unit and allowing the interpretation of a clear fragmentation pathway between the *m*/*z* 396 and 152 ions. For a full interpretation, see “Results” and Scheme 1.

Observed mass (*m*/*z*)	Atomic composition assigned	Theoretical mass (*m*/*z*)
111.0441	C6H7O2	111.0446
152.0383	C4H10NO3S	152.0381
175.1192	y″1	175.1195
204.0873	C8H14NO5	204.0872
223.0753	C7H15N2O4S	223.0752
251.0700	C8H15N2O5S	251.0701
268.0965	C8H18N3O5 S	268.0967
288.2034	y″2	288.2035
378.1333	C14H24N3O7S	378.1335
396.1432	C14H26N3O8S	396.1441
749.3781	y″7	749.3793
952.4579	y″7 + HexNAc	952.4587
1090.5752	Peptide (M + H^+^)	1090.5745
1293.6548	Peptide + HexNAc	1293.6538

The *m*/*z* 152 signal was found to possess an unusually mass-deficient accurate mass, which suggested a sulfur-containing atomic composition determined as C_4_H_10_NO_3_S for this terminal fragment, which is not the formula of any previously reported protein- or carbohydrate-derived structural unit. To help define this fragment in more detail and to provide supporting evidence for the ideas in Scheme 1, two further sets of experiments were carried out on the remaining small quantities of material: (*a*) MS/MS analysis of several of the key fragment ions above to confirm mechanistically understandable breakdown products and (*b*) experiments in the negative ion MS and MS/MS modes to look for new and complementary fragment ion information.

**FIGURE 2.**
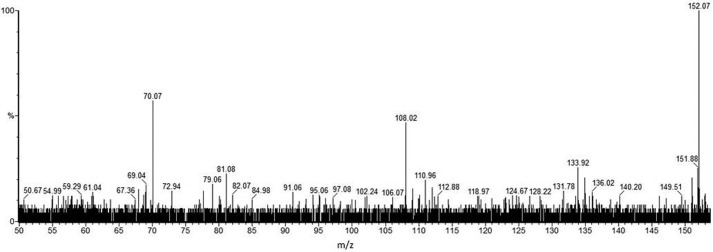
**Positive ion nanospray CAD MS/MS spectrum of *m*/*z* 152 produced via cone voltage-induced in-source fragmentation of *m*/*z* 998^2+^.** Signals at *m*/*z* 70 and 108 correspond to losses of 82 and 44 mass units, respectively, and were correlated with the low mass high resolution data from Fig. 1 to deduce atomic compositions for these ions of C_4_H_8_N and C_2_H_6_NO_2_S. This suggests two overlapping fragments derived from the *m*/*z* 152 ion (C_4_H_10_NO_3_S in Table 1), an alkylamine and a possible sulfonic acid. For a full interpretation, see “Results” and Scheme 1.

**FIGURE 3.**
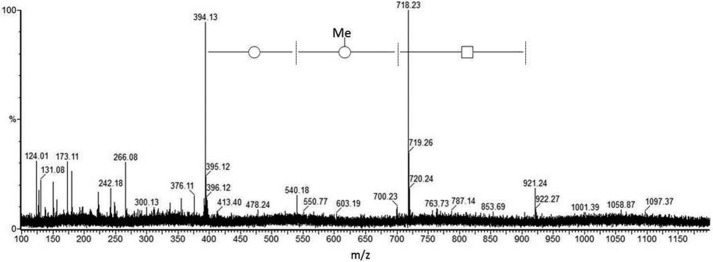
**Negative ion nanospray CAD MS/MS spectrum of *m*/*z* 996^2−^ (*m*/*z* 100–1200 mass range).** Negative ion fragmentation data complement and expand those from positive ion experiments, as here for *m*/*z* 394 (equivalent to *m*/*z* 396 in Fig. 1), *m*/*z* 266 (*m*/*z* 268), *m*/*z* 173 (*m*/*z* 175), and *m*/*z* 150 (*m*/*z* 152). Interestingly, a moderately intense low mass fragment was observed at *m*/*z* 124 (lowest mass significant fragment), and this was therefore chosen for further CAD MS/MS analysis; see Fig. 4. The highest mass signal seen at *m*/*z* 921 corresponds to the elimination of the complete sulfonated peptidylamido-glycan from the peptide backbone to leave a dehydropeptide (not observed). For a full interpretation, see “Results.”

**FIGURE 4.**
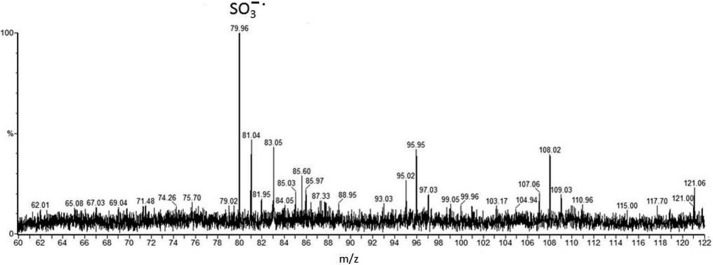
**The negative ion nanospray CAD MS/MS spectrum of *m*/*z* 124 produced via cone voltage induced in-source fragmentation of *m*/*z* 996^2−^.** The base peak (main signal) observed is present at *m*/*z* 79.96, interpreted as SO_3_^˙̄^, together with minor, less informative signals. This spectrum provided the first evidence of the presence of a sulfonic acid group in the new structure. A sample of synthetic taurine was then analyzed, showing an equivalent negative ion spectrum with base peak *m*/*z* 79.96.

CAD MS/MS of the cone voltage-induced *m*/*z* 152 from the 991^2+^ and 998^2+^ glycopeptide samples gives rise to two principal ion species seen in Fig. 2 at *m*/*z* 108 (C_2_H_6_NO_2_S) and *m*/*z* 70 (C_4_H_8_N), suggesting two overlapping component fragments (a sulfonic acid and an alkylamine) competitively derived from the *m*/*z* 152 ion (C_4_H_10_NO_3_S). The negative ion CAD MS/MS spectrum obtained for *m*/*z* 996^2−^ is shown in Fig. 3. First, these data confirm the basic structural features of the novel glycosylation inferred from the positive ion data in Fig. 1 and shown in Scheme 1 regarding expected principal fragments at *m*/*z* 921, 718, 540, 394, 266, 180, and 150, with *m*/*z* 718, for example, corresponding to X-aminodideoxyHex-deoxyHex-methyldeoxyHex**^−^**. Some other signals are derived from the carrier peptide LLDGSSTEIR, but importantly, a new fragment ion (with the equivalent not being observed in the positive ion MS/MS), is seen at *m*/*z* 124, and this was recognized as corresponding to the mass of aminoethyl-sulfonic acid, taurine (C_2_H_6_NO_3_S, M − H^−^). The MS/MS of this 996^2−^-derived signal gave rise to an MS/MS spectrum (Fig. 4) identical to that observed for a synthetic sample of taurine itself, being mainly an SO_3_^˙̄^ fragment ion base peak, thus providing strong evidence for this structural unit within the *m*/*z* 152 ion in the novel structure.

Further experiments involving subfragment MS/MS and also hydrogen/deuterium exchange to count heteroatom-linked protons were carried out to validate the structural conclusions from the mass spectrometric experiments (data not shown), but as seen in the structural summary in Scheme 1, an ambiguity nevertheless remains in the proposed novel *m*/*z* 268 unit, whereby although the amino acid amide-linked to the aminodideoxyHex would be glycine in the 991^2+^ structure, it could be either alanine or *N*-methyl glycine in the 998^2+^ structure. There are also several possible ways of arranging the R, R_1_, and R_2_ atoms in the unit incorporating the aminoethyl sulfonic acid-containing *m*/*z* 152 (*m*/*z* 180) structure, including variants containing taurine itself or cysteic acid with alkylations, to produce the necessary accurate masses observed, thus satisfying the atomic compositions determined from high resolution mass measurement. To fully characterize the R-groups in Scheme 1 and to define the stereochemistry and linkages, a scaled up preparation of flagellin was made, which was used to then allow the isolation of sufficient material for the derivation of NMR data.

##### NMR Analysis of Flagellin Glycopeptides

**FIGURE 5.**
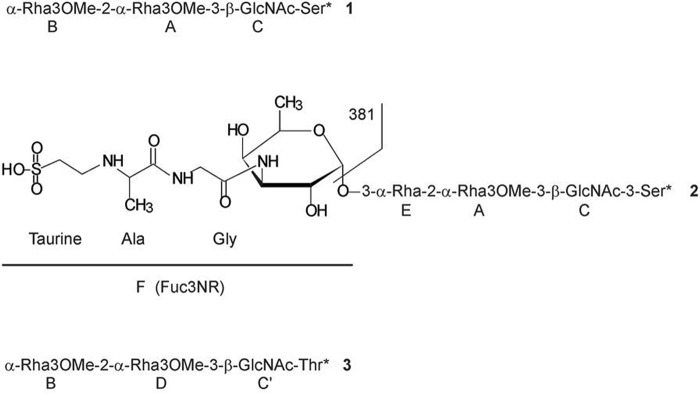
***C. difficile* R20291 flagellar glycan compounds.** Structures of isolated compounds 1, 2, and 3 are presented. *A*, *B*, *C*, *C*′, *D*, *E*, and *F below* the structures indicate the assignment of resonances presented in Fig. 6 and Tables 2 and 3. *381*, fragment identified by MS analysis in Fig. 1 (*inset*, *m*/*z* 382).

Flagellin protein (15 mg) was extensively digested with proteinase K to enable purification of sufficient quantities of glycan material with minimal peptide backbone for NMR structural analysis. This proteinase K-digested material was subjected to fractionation to obtain glycan-enriched material using size exclusion (Biogel P10) chromatography followed by Zorbax C18 reverse phase. All fractions were analyzed by ^1^H NMR to identify fractions containing glycopeptide material. Three fractions were obtained following chromatographic separation, which contained sufficient amounts of glycopeptide for further structural characterization. Fraction 21 from the initial Zorbax C18 column separation contained a mixture of two glycan species (compounds 1 and 2, Fig. 5). Fraction 23 and the reseparated fraction 12 contained compounds 2 and 3 (Fig. 5).

2D NMR spectra (COSY, TOCSY, ROESY, ^1^H-^13^C HSQC, HMBC, and HSQC-TOCSY) were recorded for all glycopeptide fractions. Spectra of fraction 21 (Table 2) contained spin systems of α-Rha3OMe (Fig. 5, *A* and *B*), β-GlcNAc (*C*), two serine residues, and one threonine residue. The position of the methyl group on Rha3OMe was determined from HMBC correlation between Me and H/C-3 of this sugar. The sequence of the monosaccharides followed from NOE and HMBC correlations between H-1 of residue B and H/C-2 of the residue A, and between H-1 of A and H/C-3 of β-GlcNAc C. H-1 of GlcNAc C showed NOE correlation to Ser* H-3 and HMBC correlation to Ser* C-3, indicating that β-GlcNAc was linked to O-3 of a serine residue (Ser*). Thus, the oligosaccharide 1 had the structure shown in Fig. 5. The purified glycopeptide contained two serine residues and one threonine residue.

**TABLE 2:** **NMR data for compound 1** NAc: 175.8; 2.11/23.7 ppm. Me: 3.44/57.2; 3.45/57.7 ppm.

Fraction 21	H/C-1	H/C-2	H/C-3	H/C-4	H/C-5	H/C-6
RhaMe A	5.08	3.97	3.56	3.50	4.02	1.25
	100.9	76.8	80.4	72.2	70.2	17.6
RhaMe B	4.89	4.27	3.45	3.46	3.75	1.29
	103.5	67.0	80.5	72.2	70.1	18.2
GlcNAc C	4.67	3.87	3.60	3.54	3.49	3.79; 3.94
	100.9	56.3	82.9	69.5	77.1	61.7
Ser*		4.36	4.17; 4.17			
	168.2	54.2	68.0			

Another glycopeptide fraction obtained after reseparation contained a mixture of compounds 2 and 3 (Fig. 5). Compound 2 contained all components of the oligosaccharide 1 without an *O*-Me group on O-3 of the terminal Rha and additionally a residue of 3-amino-3,6-dideoxy-α-galactopyranose (Fuc3N), acylated at the amino group with the unusual sulfopeptide. The position of Fuc3N at O-3 of the Rha E followed from NOE and HMBC correlations F1:E3. The glycopeptide contained three components: taurine, alanine, and glycine. The sequence of these components was determined based on the following observations. Glycine C-2 was observed at its usual place around 44 ppm, providing clear evidence that it was acylated by the alanine moiety. In contrast, the low field position of the Ala C-2 at 57.5 ppm indicated that its NH group is alkylated, (rather than if it was within a peptide, where the normal field position would be ∼51 ppm). This provides evidence that the taurine unit (deduced from the independent mass spectrometric analysis) is linked to the alanine and that glycine would be acylating N-3 of Fuc3N, supporting the interpretation of the mass spectrometric data. The sequence of the sulfopeptide would then be taurinyl-Ala-Gly, as indicated in Fig. 5 (compound 2).

**FIGURE 6.**
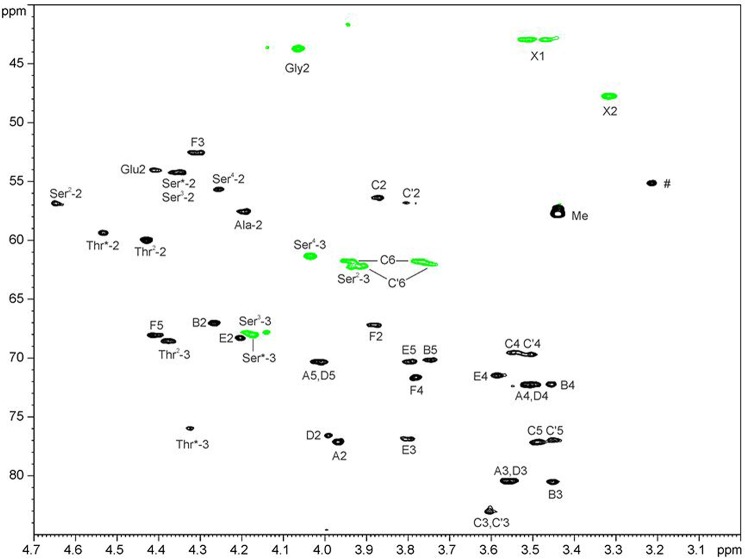
**Part of the ^1^H-^13^C HSQC spectrum of the mixture of compounds 2 and 3.** The signal *marked* # probably belongs to a structure 2 with an additional Me group, not yet localized. Amino acids *marked* with *superscript numbers* are from the peptide part, sequence not determined. Thr* and Ser* are glycosylated.

Another component of this fraction was compound 3, which had the same sugar structure as compound 1 but was linked to a threonine residue. The ^1^H and ^13^C NMR data for the glycopeptides present in fraction 21 are shown in Tables 2 and 3, and the HSQC spectrum is shown in Fig. 6.

**TABLE 3:** **NMR data for compounds 2 and 3** Atom numbering in HSO_3_CH_2_CH_2_NH-Ala (X) from Ala side. NAc: 175.8; 2.11/23.7 ppm. Me: 3.44/57.2; 3.45/57.7 ppm.

Fraction 21	H/C-1	H/C-2	H/C-3	H/C-4	H/C-5	H/C-6
RhaMe A	5.08	3.97	3.56	3.51	4.01	1.25
	101.0	77.1	80.4	72.2	70.3	17.6
Rha E	4.89	4.21	3.80	3.58	3.80	1.32
	103.2	68.2	76.9	71.4	70.2	18.3
GlcNAc C	4.66	3.87	3.60	3.54	3.48	3.77; 3.94
	100.9	56.3	83.0	69.5	77.1	61.8
Fuc3NR F	5.09	3.88	4.31	3.78	4.42	
	96.6	67.1	52.4	71.6	68.0	
Ser*		4.36	4.17; 4.17			
		54.2	68.0			
Ala	171.5	4.20	1.61			
		57.5	16.5			
Gly		4.06				
		43.7				
X	3.47; 3.51	3.32; 3.32				
	42.9	47.7				
RhaMe B	4.89	4.27	3.45	3.46	3.74	1.29
	103.2	67.0	80.5	72.2	70.1	18.2
RhaMe D	5.09	3.99	3.56	3.51	4.01	1.25
	100.9	76.5	80.4	72.2	70.3	17.6
GlcNAc C′	4.62	3.79	3.60	3.50	3.45	3.75; 3.92
	100.2	56.8	83.0	69.7	77.0	62.0
Thr*		4.53	4.32			
		59.3	75.9			

These NMR data show that the R_2_ group in Scheme 1 of the mass spectrometric interpretation is a methyl group (thus allowing an alanine assignment) to give an Ala-Gly dipeptide, with R being a hydrogen and R_1_ the ethyl sulfonic acid group giving rise to taurine in the MS/MS fragmentation. The ^13^C chemical shifts observed were in good agreement for an alkylated NH group in the alanine unit and with the shifts reported for taurine. The glycan portion of the structure, assigned mass spectrometrically as an amino-dideoxyHex-deoxyHex-methyldeoxyHex-HexNAc unit in Fig. 1 was then further assigned from the NMR data as α-Fuc3N-(1→3)-α-Rha-(1→2)-α-Rha3OMe-(1→3)-β-GlcNAc-(1→)Ser. The overall structure of the “991” peptidyl-glycan is shown as compound 2 in Fig. 5, and our mass spectrometric data suggest that the corresponding “998^2+^” peptidyl-glycan will have either alanine or *N*-methyl glycine replacing the glycine in the dipeptide portion of that structure, producing the observed 14-mass unit shift. Although the NMR spectra of compounds 2 and 3 contained additionally a minor methyl group signal, at 3.21/55.1 ppm, which may be responsible for the observed mass increase of 14 atomic mass units in the mass spectrum of some glycopeptides (“998”), we were unable to assign the position of this methyl group within the structure.

## Discussion

Glycosylation is a key modification of proteins and lipids that is often important in intermolecular and intercellular interactions. Bacterial protein glycosylation systems have come under enhanced scrutiny because of the increasing association with pathogenic species. Recent research is providing compelling evidence for protein glycosylation being central to the survival and pathogenesis of many bacteria. They have been described variously as being important in adhesion, motility, DNA uptake, biofilm formation, autoaggregation, invasion, serum resistance, immune evasion, and animal colonization (1–7, 19–25). Recently, the structure and biological role of flagellar glycosylation in the enteric opportunistic pathogen *C. difficile* 630 has been described (8, 9). Moreover, investigations into the biological role of flagellar glycosylation in the emerging hypervirulent *C. difficile* RT027 and RT023 have been undertaken and reported in our accompanying paper (26). In both *C. difficile* studies, flagella post-translational modification plays a role in motility, aggregation, and adhesion to abiotic surfaces. In the case of *C. difficile* RT027, flagella glycosylation is also involved in Caco-2 cell adhesion. In the present study, we have revealed the discovery of a unique flagellar non-reducing end peptidylamido-glycan structure on glycoproteins isolated from RT027 and other RTs (RT023, RT001, and RT106).

Following the mass spectrometric discovery of unusual post-translational modifications (PTMs) in the LC-MS and MS/MS data from tryptic digests of the FliC protein using an instrument tuned for optimal automated information-dependent acquisition, a full battery of advanced MS techniques (15–18) was first applied to characterize the novel components to the extent possible on the small quantities of protein available. These included the production of on-line high resolution MS/MS data using a 40,000 resolving power Q-TOF geometry instrument (see “Experimental Procedures”), allowing the assignment of probable atomic compositions of all fragment ions, including those clearly not derived from normal glycopeptides or previously reported PTMs. These experiments showed for the first time the presence of sulfur-containing moieties, and subsequent MS^2^ and MS^3^ data generated by the high sensitivity and mass accuracy of the Xevo Q-TOF geometry instrument (15, 16) in both positive and negative ion mode allowed the discovery of a taurine (aminoethyl-sulfonic acid) unit in the breakdown fragments of several of the precursor ion species investigated. Combining all of these data sets, together with confirmatory experiments, such as hydrogen/deuterium exchange analysis (not shown), allowed the novel structural unit containing the sulfonic acid group to be defined as shown in Scheme 1 (with composition C_4_H_10_NO_3_S). When it then became possible to isolate a sufficient amount of the des-methyl (991^2+^) variant of the structure, a detailed NMR study was used to reveal the identity of the R, R_1_, and R_2_ groups shown to give the overall PTM structure in Fig. 5. Our mass spectrometric studies on hypervirulent strains ribotype 027, 023, 106, and 001 have shown the presence of one of these structures (991^2+^ or 998^2+^) and related variants (data not shown) in each of the strains studied. The work reported here has concentrated on what was found to be the most abundant LLDGSSTEIR tryptic glycopeptide, but preliminary mass spectrometric analysis shows that similar modifications are present on at least two other flagellin peptides, QMVSSLDVALK and VALVNTSSIMSK. Only minor amounts of the respective free (non-glycosylated) peptides were found in the digests.

In contrast to this very complex glycosylation of the flagella of *C. difficile* RT027, the 630 type strain is modified with single GlcNAc residues that are substituted with an *N*-methylated threonine linked via a phosphodiester bond (8, 9). Despite the substantial differences in glycosylation, a common feature is the presence of a negatively charged functionality in the periphery of the post-translational modification, namely a sulfonate in the hypervirulent strains and a phosphoester in the 630 strain. These charged groups are likely to be involved in ionic interactions between the flagella and extracellular structures. This could explain the phenotype of *C. difficile* flagellar glycosylation knockouts, where autoaggregation, biofilm formation, and adhesion to Caco-2 cells are reduced (9, 26).

An increasing number of reports on flagellar glycosylation on Gram-positive and Gram-negative bacterial pathogens have been published (10, 27). In comparison with Gram-negative bacteria, the reports on flagellar glycosylation on Gram-positive are limited. Among Gram-positive bacteria, *Clostridium* spp. are the most characterized (8, 9, 28–30). There are two other genera of Gram-positive pathogens with *O*-glycosylated flagella: *Listeria* and *Paenibacilla. Listeria monocytogenes* is glycosylated at up to six sites per monomer with a single β-*O*-linked GlcNAc residue (11). *Paenibacilla* has a flagella modified with an *O*-linked trisaccharide composed of one hexose and two *N*-acetyl-hexosamine residues at three sites of glycosylation (13). In the case of Gram-negative bacteria, the diversity of flagellar glycosylation moieties is remarkable. Flagellins of many Gram-negative bacterial pathogens have a single pseudaminic or legionaminic acid residue at each of their *O*-glycosylation sites (10). Both of these sugars exhibit considerable diversity due to differences in acyl functionalities. Notably, some are acylated with amino acids. Thus, glycine and *N*-acetyl glutamine have each been observed as ester-linked substituents of pseudaminic acid in the flagella of *Aeromonas caviae* and *Campylobacter jejuni*, respectively (31). However, there are no reports of peptidyl substituents on glycans from Gram-negative organisms. In addition, amino acid substituents have not previously been observed on the glycans of Gram-positive pathogens. Therefore, our discovery that the *C. difficile* RT027 flagella are modified by a peptidylamido-sugar moiety is a unique finding within both Gram-positive and Gram-negative bacteria.

Another structural component, identified in the present study as a taurine-like non-reducing end unit, is unique in flagella glycosylation from bacteria. This unit might be used by RT027 strains as a strategy to evade the host immune system because taurine is reported to have a key role in the regulation of the innate immune response (32).

In some bacterial pathogens, such as the enteric pathogen *C. jejuni*, glycosyltransferases and glycan biosynthetic genes are situated adjacent to biosynthetic flagellar genes (33). For *C. difficile* type B flagellin, it appears that rhamnose biosynthetic genes (CDR20291_0223–0226), which are similar to *rmlD*, *rmlA*, *rmlC*, and *rmlB*, lie upstream of *fliC* and are probably involved in the biosynthesis of the rhamnose moieties modifying the *C. difficile* RT027 flagella (26). Within the locus immediately downstream of *fliC* in addition to the three putative flagellar glycosyltransferase genes, there are a number of biosynthetic genes (CDR20291_0244–0247) that appear to be responsible for synthesis of the novel terminal moiety (26). These biosynthetic genes are present in the genomes of both RT027 and RT023 strains. The amino acid similarity of CDR20291_0247 to FdtB from *A. thermoaerophilus* suggests that this gene product is probably involved in the production of the 3-amino-3,6-dideoxy-α-galactopyranose (Fuc3N) monosaccharide. Previous work using *Aneurinibacillus thermoaerophilus* and *Xamthomonas campestris* enzymes have shown that RmlA and RmlB catalyze the first two steps for Fuc3N biosynthesis (34) by producing the substrate dTDP-6-deoxy-d-xylohex-4-ulose. Optimally, this substrate would be converted to dTDP-6-deoxy-d-xylohex-3-ulose by FdtA; however, it has been demonstrated that incubation of the 4-keto product with FdtB and cofactors will yield a moderate level of conversion to dTDP-Fucp3N in the absence of FdtA isomerase (34). It has been suggested that this may be due to production of 3-keto substrate via non-enzymatic processes (35). Because no homolog of FdtA appears to be present in the *C. difficile* R20291 type B post-translational modification locus, it appears that this may be the mechanism whereby limited amounts of Fuc3N can be produced in *C. difficile* and incorporated into the flagellar glycan. The heterogeneity observed in flagellin glycan composition (sulfonated peptidylamido-glycan structure and truncated methylated trisaccharide (Rha-Rha-GlcNAc) structure) might be explained by the inefficient production of Fuc3N from dTDP-6-deoxy-d-xylohexose-4-ulose by FdtB. Limiting amounts of Fuc3N would prevent the synthesis of the full flagellar glycan. In addition to the FdtB homolog, there exists a putative acyltransferase (CDR20291_0244), which is a candidate for acylating the amino group of the fucosamine moiety. Downstream of CDR20291_0244 lie CDR20291_0245 and CDR20291_0246, which are co-transcribed with the glycosyltransferase genes and encode a putative d-alanine ligase and alanine dehydrogenase, respectively. These genes are probably involved in the biosynthesis of the peptidyl moiety of this novel flagellar glycan (26).

This study reveals a unique flagellar glycosylation structure in the bacterial pathogen *C. difficile* hypervirulent RT027 strains, which could provide the organism with a novel strategy to escape the immune system and be more virulent. Furthermore, this work highlights the diversity of glycans modifying flagella in other hypervirulent *C. difficile* RTs, such as RT023, RT001, and RT106, which could suggest different strategies of *C. difficile* to evade the immune system.

## Experimental Procedures

### 

#### 

##### Bacterial Growth and Cultures

*C. difficile* strains used in this study are shown in Table 4. Strains were routinely grown on Brazier's CCEY agar (BioConnection, Leeds, UK) containing 4% (w/v) egg yolk, 250 mg/ml cycloserine, 8 mg/ml cefoxitin (Bioconnections), and 1% defibrinated horse blood (TCS Biosciences, Buckingham, UK), in blood agar (Oxoid, Hampshire, UK) and in BHI (brain heart infusion medium (Oxoid)) and BHIS (BHI supplemented with 0.5% (w/v) yeast (Sigma, Gillingham, UK), 0.1% l-cysteine (Sigma)) broth or agar. All cultures were incubated at 37 °C overnight in a Don Whitney MG500 anaerobic work station (Don Whitney Scientific Ltd.).

**TABLE 4:** ***C. difficile* strains used in this study**

Strains	Characteristics	Source
R20291	PCR ribotype 027, isolated from an outbreak in 2004–2005	Aylesbury, UK
CD196	Ribotype 027, isolated from a pseudomembranous colitis case	France, 1985
BI-16	PCR ribotype 027, isolated from an outbreak in 2004	Augusta, GA
CD305	PCR ribotype 023, isolated from an outbreak in 2011	Barts Hospital, UK
CD1426	PCR ribotype 023, isolated from an outbreak in 2010	Queens Hospital Remford, UK
CD1714	PCR ribotype 023, isolated from an outbreak in 2011	Whipp's Cross Hospital, UK
106-01	PCR ribotype 106, isolated from an outbreak in 2008	Fatal case, Glasgow, UK
001-01	PCR ribotype 001, isolated from an outbreak in 2008	Paisley, UK
001-07	PCR ribotype 001, isolated from an outbreak in 2008	Edinburgh, UK

##### Protein Isolation and Digestion

Bacteria were grown on BHIS overnight, at 37 °C. *C. difficile* strains were harvested, washed in phosphate-buffered saline, and resuspended in a 1:100 volume of low pH glycine (0.2 m glycine-HCl, pH 2.2) and incubated at room temperature for 30 min with gentle shaking. The cells were removed by centrifugation at 4 °C, and the supernatant was neutralized with the addition of 2 m Tris to a pH of 7–8. Flagellin preparations were analyzed by 12% SDS-PAGE Novex® NuPAGE® Tris-glycine SDS-PAGE. The separated proteins were stained with Coomassie Blue in accordance with standard techniques. Flagellin bands were excised, lyophilized, and digested with trypsin (EC 3.4.21.4; Promega) overnight. Peptides were extracted from gel pieces and studied by LC-MS/MS analysis.

##### Mass Spectrometry

Positive ion Q-TOF technology (15, 16) was used to study the tryptic digests of proteins isolated from the various strains of *C. difficile* ribotypes in LC-MS and MS/MS experiments conducted on several instruments. A Sciex QStar Pulsar 1 instrument with LC Packings nano-LC using a C-18 nanocapillary (75 μm × 15-cm Pepmap) column eluting with a 0.05% formic acid, 5–95% acetonitrile gradient over 90 min, and information-dependent acquisition was used for the low resolution (5000 resolving power) analysis of digest mixtures. A Waters XevoG2 instrument with Acquity microbore UPLC, using a C-18 (1 × 50-mm BEH) column eluting with a 0.1% formic acid, 0–85% acetonitrile gradient over 40 min, with data-dependent acquisition, was used for detailed positive and negative ion MS*^n^* and cold isotope-labeling experiments, and a Waters Synapt G2-S, with nanoAcquity UPLC and a 75 μm × 15-cm BEH column and gradient elution with 0.05% formic acid, 5–95% acetonitrile over 45 min, and data-dependent acquisition, was used for the collection of the high resolution (40,000 resolving power) MS/MS data from which atomic compositions could be calculated. MS^3^ data were generated on the XevoG2 instrument on various fragment ions of interest by nanospray of 1–2 μl of LC-collected fractions loaded into borosilicate needles, using high cone voltage to achieve source fragmentation and then passing the fragment ions of interest into the collision cell for CAD MS/MS.

Negative ion mass spectrometry was used to study collected fractions of interest. These were adjusted to basic pH using 10% ammonia, and then MS^3^ data were generated on the XevoG2 instrument on various fragment ions of interest by nanospray as described above.

##### NMR Glycan Structural Analysis

Large scale preparation of flagellin was performed by growing *C. difficile* R20291 overnight on 40 BHIS agar plates at 37 °C in an anaerobic chamber. Cells were harvested into sterile distilled water (dH_2_O), and cells were vortexed for 3 min to release flagella filaments from cells. Bacterial cells were removed by centrifugation at 13,000 rpm in an Eppendorf centrifuge for 5 min (twice). The supernatant was collected, pooled, and passed through a 13-mm PVDF filter. This sterile supernatant was then centrifuged at 55,000 rpm for 1 h at 4 °C in a Beckman Optima TLX Ultracentrifuge using a Beckman TLA 120.2 rotor. The pelleted material was resuspended in dH_2_O, and the ultracentrifugation step was repeated. The supernatant was discarded, and the pellet was resuspended in dH_2_O. Flagellin protein was obtained from multiple bacterial growths (>10) to give a total of 10 mg of flagellin protein at a concentration of 0.4 mg/ml (as determined by the Bio-Rad protein assay). To obtain glycan material devoid of protein backbone for structural analysis, flagellin (10 mg) was digested with proteinase K at a ratio of 1:1 (Sigma) in 10 mm Na_2_PO_4_, pH 7.6, at 37 °C for 48 h. The proteinase K-digested material was lyophilized and resuspended in dH_2_O, and the sample was fractionated by gel filtration on a Biogel P10 column (2.5 × 80 cm, 1% acetic acid, refractive index detector). Each fraction was analyzed by ^1^H NMR, and the glycopeptide-containing fraction was then applied to a Zorbax C18 column in a 0.1% TFA, 80% acetonitrile gradient with a UV detector at 220 nm, and fractions were collected and reexamined by ^1^H NMR for the presence of glycan. Separation was then repeated with selected fractions using a gradient from 4 to 20% of 80% acetonitrile over 1 h. All fractions were then analyzed by NMR.

##### NMR Spectroscopy

NMR experiments were carried out on a Bruker AVANCE III 600 MHz (^1^H) spectrometer with a 5-mm Z-gradient probe with acetone internal reference (2.225 ppm for ^1^H and 31.45 ppm for ^13^C) using standard pulse sequences cosygpprqf (gCOSY), mlevphpr (TOCSY, mixing time 120 ms), roesyphpr (ROESY, mixing time 500 ms), hsqcedetgp (HSQC), hsqcetgpml (HSQC-TOCSY, 80-ms TOCSY delay), and hmbcgplpndqf (HMBC, 100-ms long range transfer delay). Resolution was kept <3 Hz/point in F2 in proton-proton correlations and <5 Hz/point in F2 of H-C correlations. The spectra were processed and analyzed using the Bruker Topspin version 2.1 program.

## Author Contributions

E. Valiente, A. D., B. W. W., and H. R. M. designed research; E. Valiente purified samples for mass spectrometry; L. B., M. P., P. H., D. B., F. S., A. D., and H. R. M. performed mass spectrometric analysis and data interpretation; E. Valiente, A. A., K. F., and S. T. purified samples for NMR; E. Vinogradov, A. A., A. F.-P., K. F., S. T., and S. M. L. performed NMR analysis and data interpretation. L. B. and S. M. L. prepared figures; L. B., M. P., E. Vinogradov, S. T., S. M. L., B. W. W., A. D., and H. R. M. wrote the manuscript; all authors edited the paper.

## Footnotes

4 The abbreviations used are:

RT: ribotype
HMBC: heteronuclear multiple-bond correlation
Fuc3N: 3-amino-3,6-dideoxy-α-galactopyranose
GlcNAc: *N*-acetylglucosamine
HexNAc: *N*-acetylhexosamine
HSQC: heteronuclear single quantum correlation
PTM: post-translational modification
CAD: collisionally activated decomposition
dH_2_O: distilled H_2_O.
